# Prevalence of Adhesive Capsulitis in Patients With Type 2 Diabetes Mellitus: A Single-Center Cross-Sectional Study From Pakistan

**DOI:** 10.7759/cureus.70675

**Published:** 2024-10-02

**Authors:** Nauman Ismat Butt, Muhammad Sohail Ajmal Ghoauri, Umaima Waris, Dur Sabeh, Fahad Qaisar, Ali Imran

**Affiliations:** 1 Internal Medicine and Rheumatology, Azra Naheed Medical College, Superior University, Lahore, PAK; 2 Medicine and Neurology, Bahawal Victoria Hospital, Quaid-e-Azam Medical College, Bahawalpur, PAK; 3 Medicine, Azra Naheed Medical College, Superior University, Lahore, PAK; 4 Medicine, Bahawal Victoria Hospital, Quaid-e-Azam Medical College, Bahawalpur, PAK

**Keywords:** adhesive capsulitis, diabetes mellitus type 2, frozen shoulder, glycated hemoglobin (hba1c), shoulder trauma

## Abstract

Background

An inflammatory condition leading to stiffness and pain in the shoulder joint, adhesive capsulitis is associated with conditions such as diabetes mellitus, cervical spondylosis, thyroid dysfunction, autoimmune rheumatic diseases, and shoulder injury due to trauma, fracture, rotator cuff tear, surgery, or immobilization. Adhesive capsulitis may affect a notable proportion of the diabetic population. There are numerous studies that show that patients with type 2 diabetes mellitus are not only at higher risk of developing adhesive capsulitis but also suffer poor outcomes despite treatment, especially in patients with long-standing diabetes mellitus. Furthermore, there is significant variation in data regarding the prevalence of adhesive capsulitis in Pakistani patients with type 2 diabetes mellitus.

Objective

To determine the prevalence of adhesive capsulitis among patients with type 2 diabetes mellitus presenting to a tertiary care hospital in Bahawalpur, Pakistan.

Methods

The present observational cross-sectional study was carried out at the Department of Medicine, Bahawal Victoria Hospital, Quaid-e-Azam Medical College, Bahawalpur, Pakistan, from February 2024 to August 2024. Type 2 diabetes mellitus was labeled by HbA1c of more than 7.0%, or two random blood glucose levels of 200 mg/dL or more, or an existing diagnosis of diabetes mellitus, and/or use of anti-hyperglycemic therapy. Adhesive capsulitis was diagnosed clinically on the basis of history (gradual onset shoulder pain with limitation of movements) and examination (reduction in active and passive range of motion (ROM) of the shoulder, especially abduction, internal rotation, and external rotation) in the absence of significant abnormalities on shoulder X-ray. After ethical approval and obtaining informed consent, 430 patients with type 2 diabetes mellitus were included in the study using a non-probability consecutive sampling technique. Demographic information, diabetes control, and HbA1c levels were noted, and the patients were assessed for adhesive capsulitis. All the data was recorded and entered into IBM SPSS Statistics for Windows, Version 23 (Released 2015; IBM Corp., Armonk, NY, USA), for analysis.

Results

Having a female preponderance (266, or 61.9%), the mean age of the participants was 54.0 ± 13.1 years. With regard to occupational status, 126 (29.3%) had a sedentary occupation, 45 (10.5%) were unemployed, and 259 (60.2%) had a non-sedentary occupation. The mean diabetes duration was 6.4 ± 5.3 years, and the majority of patients had poor diabetes control (322, or 74.9%). Adhesive capsulitis was present in 61 (14.2%) patients with type 2 diabetes mellitus. On stratification, no significant statistical association of adhesive capsulitis was seen with gender (p-value: 0.075), age (p-value: 0.465), occupation (p-value: 0.056), diabetes duration (p-value: 0.118), or diabetes control (p-value: 0.090).

Conclusion

Adhesive capsulitis was not an uncommon finding in our study, reported in almost one-fifth of the 430 patients enrolled. We recommend that treating physicians screen diabetic patients for adhesive capsulitis so that proper pain relief, physiotherapy, and rehabilitation may be provided timely and efficiently, thereby reducing morbidity and improving the quality of life for such patients.

## Introduction

An inflammatory condition leading to stiffness and pain in the shoulder joint, adhesive capsulitis is also referred to as a frozen shoulder. Depending on the cause, adhesive capsulitis is classified as primary or secondary. Being idiopathic with a gradual onset, primary adhesive capsulitis is associated with conditions such as diabetes mellitus, cervical spondylosis, thyroid dysfunction, autoimmune rheumatic diseases, Parkinson's disease, and certain drugs, such as fluoroquinolones and barbiturates [1,2]. Secondary adhesive capsulitis is usually a consequence of shoulder injury due to trauma, fracture, rotator cuff tear, surgery, or immobilization [3,4]. Having a prevalence of up to 5% in the general population, adhesive capsulitis is slightly more common in women than in men (1.4:1) and often affects non-dominant individuals [5]. The timeline of adhesive capsulitis development and progression can be divided into an initial inflammatory phase characterized by pain and tenderness, followed by a fibrotic phase highlighted by stiffness and limitations in range of motion (ROM), and finally, a regression phase in which the shoulder thaws and mobility gradually improves [3].

The diagnosis of adhesive capsulitis relies on the American Academy of Orthopedic Surgeons' definition. Based on clinical history and examination, the gradual onset of global limitations in the movements of the shoulder, in the absence of significant radiographic abnormalities, helps to establish the diagnosis [6,7]. Adhesive capsulitis may be seen in up to 20% of the diabetic population [3]. A major health concern on the rise, it has been estimated that diabetes mellitus will affect 438 million people globally by 2030, with a major disease burden affecting Asian populations [8]. Despite advances in treatment, complications and comorbidities of diabetes are still high, leading to significant morbidity, disability, and burden on constrained healthcare resources in resource-limited countries such as Pakistan [9].

There are numerous studies that show that patients with type 2 diabetes mellitus are not only at higher risk of developing adhesive capsulitis, but also suffer poor outcomes despite treatment, especially in patients with long-standing diabetes mellitus [10,11]. Furthermore, there is significant variation in data regarding the prevalence of adhesive capsulitis in Pakistani patients with type 2 diabetes mellitus. Adhesive capsulitis was seen in 24.9% of patients with diabetes mellitus in the study by Ahmad et al. [12]. Seher et al. reported adhesive capsulitis to be present in 66.67% of patients with diabetes mellitus compared to 9.33% in non-diabetics [13]. In the study by Inayat et al., 43.1% of diabetic patients had adhesive capsulitis [14]. Therefore, we conducted the present study to determine the prevalence of adhesive capsulitis among type 2 diabetes mellitus patients presenting to a tertiary care hospital in Bahawalpur, Pakistan, so that timely diagnosis and prompt management of these patients may lead to a reduction in associated morbidity and disability.

## Materials and methods

To determine the prevalence of adhesive capsulitis among patients with type 2 diabetes mellitus, the present observational cross-sectional study was carried out at the Department of Medicine, Bahawal Victoria Hospital, Quaid-e-Azam Medical College, Bahawalpur, Pakistan, from February 2024 to August 2024. Type 2 diabetes mellitus was labeled by HbA1c of more than 7.0%, or two random blood glucose levels of 200 mg/dL or more, or an existing diagnosis of diabetes mellitus, and/or use of anti-hyperglycemic therapy. Adhesive capsulitis was diagnosed clinically on the basis of history (gradual onset shoulder pain with limitation of movements) and examination (reduction in both active and passive ROM of the shoulder, especially abduction, internal rotation, and external rotation) in the absence of significant abnormalities on shoulder X-ray [5].

Keeping a margin of error of 5% and a 95% confidence interval, a sample size of 377 was calculated using the expected frequency of adhesive capsulitis as 43.1% in patients with type 2 diabetes mellitus [14]. However, to increase the strength of our study, we included 430 patients with type 2 diabetes mellitus to assess the prevalence of adhesive capsulitis. Patients with neck pain or stiffness, autoimmune rheumatic diseases, thyroid dysfunction, chronic/end-stage kidney disease, Parkinson's disease, and chronic liver disease; patients with a history of rotator cuff tendonitis, shoulder surgery, and arm amputation or fracture; and pregnant women were excluded from the study. The rationale was to exclude patients having medical conditions associated with adhesive capsulitis and patients with secondary adhesive capsulitis due to surgery or fracture, so that only patients with adhesive capsulitis and type 2 diabetes mellitus would be studied. Shoulder X-rays were done to rule out other causes of shoulder pain and ROM limitations, such as destructive arthropathy, fracture, and calcific tendonitis, which have specific radiographic abnormalities related to them. However, an X-ray would be normal in adhesive capsulitis, and this helps to differentiate it from the other causes.

After ethical approval and obtaining informed consent, 430 patients with type 2 diabetes mellitus were included in the study using a non-probability consecutive sampling technique from February 2024 to August 2024. Demographic information, including age, gender, and occupation (type of work and job hour details), was asked of each patient by a medical professional and recorded accordingly. Sedentary occupation was defined by jobs that involve minimal physical activity, such as office or desk jobs requiring employees to sit for more than six hours per day, stand or walk less than two hours per day, and lift or carry no more than 10 pounds (4.5 kg) at a time [15,16]. Medical records of each patient were assessed for diabetes duration, diabetes control, and HbA1c levels. Good diabetes control was defined as an HbA1c of less than or equal to 7.0% on anti-hyperglycemic therapy, whereas poor diabetes control was defined as an HbA1c greater than 7.0%. All patients were then asked for medical history regarding symptoms of adhesive capsulitis, after which an examination was done. In patients having shoulder pain and ROM limitation, X-rays of the shoulder were performed to look for radiographic abnormalities, the absence of which indicated adhesive capsulitis.

All the data was recorded and entered using IBM SPSS Statistics for Windows, Version 23 (Released 2015; IBM Corp., Armonk, NY, USA), for analysis. Mean and standard deviation were calculated for quantitative variables such as age, diabetes duration, and HbA1c levels. Percentage and frequency were generated for qualitative variables such as gender, occupation, diabetes control, and adhesive capsulitis. Cumulative percent was used to determine cutoffs for age and diabetes duration quartiles. Effect modifiers and confounders were controlled through stratification, and the Chi-square test was applied post-stratification, using a p-value of less than 0.05 as significant.

## Results

A total of 430 patients with type 2 diabetes mellitus enrolled in the study, having a female preponderance (266, or 61.9%). The mean age was 54.0 ± 13.1 years, with 216 (50.2%) patients aged 55 years or more, as shown in Table 1. With regard to occupation, 126 (29.3%) had a sedentary occupation, 45 (10.5%) were unemployed, and 259 (60.2%) had a non-sedentary occupation. The mean diabetes duration was 6.4 ± 5.3 years, with 257 (59.8%) patients having diabetes for five years or more, as shown in Table 1. The mean HbA1c (%) was 9.8 ± 2.5, and the majority of patients had poor diabetes control (322, or 74.9%).

**Table 1:** Clinical and demographic variables of the patients (n = 430)

Clinical and demographic variables		Frequency (n)	Percent (%)
Gender	Female	266	61.9
	Male	164	38.1
Age	54 years or less	214	49.8
	55 years or more	216	50.2
Occupation	Non-sedentary	259	60.2
	Sedentary	126	29.3
	Unemployed	45	10.5
Diabetes duration	4 years or less	173	40.2
	5 years or more	257	59.8
Diabetes control	Good/controlled	108	25.1
	Poor/uncontrolled	322	74.9
Adhesive capsulitis	Absent	369	85.8
	Present	61	14.2

In the present study, adhesive capsulitis was present in 61 (14.2%) patients with type 2 diabetes mellitus. Out of the 61 patients with adhesive capsulitis, 54 (88.5%) had unilateral involvement, and only three (4.9%) had a prior history of shoulder trauma, as demonstrated in Table 2. Out of seven patients with bilateral adhesive capsulitis, poor diabetes control was seen in six (85.7%) patients. Among the 54 patients with unilateral involvement, 45 (83.3%) had poor diabetes control. The site of involvement and diabetes control did not demonstrate any statistical significance (p-value: 0.235). Table 3 depicts the stratification of data with regard to adhesive capsulitis. There was no significant statistical association of adhesive capsulitis with gender (p-value: 0.075), age (p-value: 0.465), occupation (p-value: 0.056), diabetes duration (p-value: 0.118), or diabetes control (p-value: 0.090).

**Table 2:** Site and trauma history in patients with adhesive capsulitis (n = 61)

Clinical variables	Frequency (n)	Percent (%)
Site involvement		
Bilateral	7	11.5
Unilateral	54	88.5
Shoulder trauma history		
Present	3	4.9
Absent	58	95.1

**Table 3:** Stratification of data with regards to adhesive capsulitis (n = 430)

Clinical and demographic variables	Adhesive capsulitis		p-value
	Absent	Present	
Gender			0.075
Female	222 (83.5%)	44 (16.5%)	
Male	147 (89.6%)	17 (10.4%)	
Age			0.465
54 years or less	181 (84.6%)	33 (15.4%)	
55 years or more	188 (87.0%)	28 (13.0%)	
Occupation			0.056
Non-sedentary	215 (83.0%)	44 (17.0%)	
Sedentary	116 (92.1%)	10 (7.9%)	
Unemployed	38 (84.4%)	7 (15.6%)	
Diabetes duration			0.118
4 years or less	154 (89.0%)	19 (11.0%)	
5 years or more	215 (83.7%)	42 (16.3%)	
Diabetes control			0.090
Good/controlled	98 (90.7%)	10 (9.3%)	
Poor/uncontrolled	271 (84.2%)	51 (15.8%)	

In a logistic regression analysis assessing the impact of various clinical predictors on adhesive capsulitis, only occupation emerged as statistically significant (p-value: 0.033), with an odds ratio of 2.871. While gender (B = -1.257; Exp(B) = 0.285) and diabetes control (B = -0.546; Exp(B) = 0.579) suggested decreased odds of adhesive capsulitis, neither reached statistical significance (p-values: 0.137 and 0.143, respectively). Additionally, age (B = -0.420; Exp(B) = 0.657) and diabetes duration (B = 0.453; Exp(B) = 1.573) showed trends toward lower and higher odds, respectively, but were also not statistically significant (p-values: 0.150 and 0.140, respectively). The constant (B = -3.522) was highly significant (p < 0.001), indicating a strong baseline effect. Overall, these findings suggest that, while occupation significantly influences the odds of adhesive capsulitis, other variables did not demonstrate meaningful associations, as shown in Table 4.

**Table 4:** Logistic regression analysis assessing the impact of various clinical predictors on adhesive capsulitis

Variables	Beta coefficient (B)	SE	Wald	df	p-value	Exp(B)	95% CI for Exp(B)	
							Lower	Upper
Gender	-1.257	0.845	2.212	1	0.137	0.285	0.054	1.491
Age	-0.420	0.292	2.070	1	0.150	0.657	0.371	1.164
Occupation	1.055	0.495	4.534	1	0.033	2.871	1.088	7.577
Diabetes duration	0.453	0.307	2.182	1	0.140	1.573	0.862	2.871
Diabetes control	-0.546	0.372	2.150	1	0.143	0.579	0.279	1.202

## Discussion

Diagnosing adhesive capsulitis is based predominantly on clinical history and physical examination; radiographic studies are performed only to exclude the differential diagnoses [3,17]. Adhesive capsulitis can lead to a negative impact on work productivity, quality of life, and the mental health of patients [17,18]. It is, therefore, necessary to diagnose and manage adhesive capsulitis in a timely manner so that its associated disability and morbidity may be reduced. Often regarded as a self-limiting disease that resolves between one and three years after onset, adhesive capsulitis may lead to long-lasting symptoms in almost 50% of patients [3]. The prevalence of adhesive capsulitis is reported to be 3-5% in the general population, but this is as high as 20% in the diabetic population [3,17]. A positive link between HbA1c and increasing age has been reported with adhesive capsulitis in diabetics [19,20].

In the present study, adhesive capsulitis was not an uncommon finding, reported in almost one-fifth (14.2%) of the 430 patients with type 2 diabetes mellitus enrolled. This is quite low when compared to other studies conducted in Pakistan. Adhesive capsulitis was seen in 24.9% and 66.67% of patients with diabetes mellitus by Ahmad et al. and Seher et al., while it was seen in 9.3% of non-diabetics [12,13]. Adhesive capsulitis was associated with increasing age and prolonged duration of diabetes [12]. It was more common in females and patients aged 46-55 years [13]. In the study by Inayat et al., 43.1% of diabetic patients had adhesive capsulitis, which was more frequent in females and patients with poor diabetes control, insulin dependence, and a positive family history of adhesive capsulitis [14]. In the present study, no significant statistical association of adhesive capsulitis was seen with gender, age, diabetes duration, or diabetes control. Furthermore, in logistic regression analysis, occupation significantly influenced the odds of adhesive capsulitis, but other variables did not demonstrate meaningful associations in our study. This disparity in our results and those of other Pakistani studies may be attributable to regional and ethnic differences, family history, mode of diagnosis (clinical versus radiography), anti-hyperglycemic therapy, or insulin use by the patients.

The treatment of adhesive capsulitis relies on symptomatic relief and improving shoulder ROM. Non-steroidal anti-inflammatory drugs (NSAIDs) have been shown to reduce pain in the initial phase [5,21]. Physical therapy, in the form of stretching, gentle ROM exercises, and gradual graded resistance training, helps to control pain and improve shoulder mobility [5,22]. However, vigorous rehabilitation should be avoided, as it can cause worsening of symptoms. In the short term, oral steroids provide pain relief, but the benefits usually last only for a few weeks. Intra-articular steroid injections and hydrodilatation help to reduce pain, increase shoulder ROM, and improve shoulder function [5,23]. In patients refractory to these maneuvers, manipulation of the shoulder under anesthesia, arthroscopic capsular release, and open capsular release have been tried and tailored according to patient specifics, underlying cause, and co-morbid conditions [5,24,25].

The differential diagnosis of shoulder pain and ROM limitation is wide and includes conditions such as fracture, cervical radiculopathy, rotator cuff tendinitis, bicep tendinopathy, calcific tendinitis, acromioclavicular joint arthrosis, glenohumeral arthritis, and polymyalgia rheumatica [26,27]. Detailed history and physical examination help to point toward adhesive capsulitis, which is usually diagnosed clinically. Shoulder radiography will be normal in adhesive capsulitis and may be done to rule out alternate diagnoses. However, further laboratory tests should be done in patients with non-specific and/or additional features to investigate any underlying cause. Patients with adhesive capsulitis typically present to their primary caregiver, and referral to a rheumatologist, orthopedic specialist, or pain management specialist may be appropriate. It has been reported that early detection of adhesive capsulitis favors a better outcome, while delay in detection leads to a worse prognosis [14]. Accurate record-keeping, effective communication, and timely referrals also aid in improving outcomes [5]. Up to 60% of patients may continue to experience persistent symptoms despite conservative treatment, and 20% of patients with adhesive capsulitis may suffer from long-term disability [5]. It is therefore pertinent to screen diabetic patients for adhesive capsulitis and perform shoulder X-rays in a timely manner to exclude other causes, so that prompt management may be initiated. Our study aims to highlight this association of diabetes mellitus with adhesive capsulitis, in addition to the need to screen diabetic patients for adhesive capsulitis and raise awareness regarding it.

There are certain limitations to our study. Even though we had an adequate sample size of 430 patients, our study was based in a single center. Furthermore, we did not study the effect of factors such as family history and anti-diabetic medications, including insulin, smoking, and alcohol use. Patients with other conditions associated with adhesive capsulitis were excluded from our study, making it difficult to comment on the interplay of co-morbid diseases and genetic and environmental factors in the etiology of adhesive capsulitis. Furthermore, we did not study the treatment given and the outcomes of the patients with adhesive capsulitis. The results of our study may not be applicable to the general population. Therefore, further multi-center studies should be conducted to document the association and role of adhesive capsulitis in type 2 diabetes mellitus among the Pakistani population and to investigate the factors related to it. A potentially treatable condition, adhesive capsulitis may negatively affect quality of life. Timely diagnosis and management can result in a decrease in disability and morbidity, leading to improved quality of life for these patients.

## Conclusions

Adhesive capsulitis was not an uncommon finding in our study, reported in almost one-fifth (14.2%) of the 430 patients with type 2 diabetes mellitus enrolled. Furthermore, among the 61 patients with adhesive capsulitis, unilateral involvement was seen in 88.5%, while 4.9% reported a prior history of shoulder trauma. On stratification, adhesive capsulitis had no significant statistical association with gender, age, occupation, diabetes duration, or diabetes control. A potentially treatable condition, adhesive capsulitis may negatively affect quality of life. Timely diagnosis and management can result in a decrease in disability and morbidity, leading to improved quality of life for these patients. We recommend that treating physicians screen diabetic patients for adhesive capsulitis so that proper pain relief, physiotherapy, and rehabilitation may be provided timely and efficiently, thereby reducing morbidity and improving the quality of life for such patients. Furthermore, awareness programs should also be conducted to educate diabetic patients regarding the signs and symptoms of adhesive capsulitis.
